# Identification and characterization of large DNA deletions affecting oil quality traits in soybean seeds through transcriptome sequencing analysis

**DOI:** 10.1007/s00122-016-2725-z

**Published:** 2016-05-14

**Authors:** Wolfgang Goettel, Martha Ramirez, Robert G. Upchurch, Yong-qiang Charles An

**Affiliations:** US Department of Agriculture, Agricultural Research Service, Midwest Area, Plant Genetics Research Unit, Donald Danforth Plant Science Center, 975 N. Warson Rd, St. Louis, MO 63132 USA; US Department of Agriculture, Agricultural Research Service, Soybean and Nitrogen Fixation Research, 2417 Gardner Hall, Raleigh, NC 27695 USA

## Abstract

****Key message**:**

**Identification and characterization of a 254-kb genomic deletion on a duplicated chromosome segment that resulted in a low level of palmitic acid in soybean seeds using transcriptome sequencing.**

**Abstract:**

A large number of soybean genotypes varying in seed oil composition and content have been identified. Understanding the molecular mechanisms underlying these variations is important for breeders to effectively utilize them as a genetic resource. Through design and application of a bioinformatics approach, we identified nine co-regulated gene clusters by comparing seed transcriptomes of nine soybean genotypes varying in oil composition and content. We demonstrated that four gene clusters in the genotypes M23, Jack and N0304-303-3 coincided with large-scale genome rearrangements. The co-regulated gene clusters in M23 and Jack mapped to a previously described 164-kb deletion and a copy number amplification of the *Rhg1* locus, respectively. The coordinately down-regulated gene clusters in N0304-303-3 were caused by a 254-kb deletion containing 19 genes including a *fatty acyl*-*ACP thioesterase B gene* (*FATB1a*). This deletion was associated with reduced palmitic acid content in seeds and was the molecular cause of a previously reported nonfunctional *FATB1a* allele, *fap*_*nc*_. The M23 and N0304-304-3 deletions were located in duplicated genome segments retained from the *Glycine*-specific whole genome duplication that occurred 13 million years ago. The homoeologous genes in these duplicated regions shared a strong similarity in both their encoded protein sequences and transcript accumulation levels, suggesting that they may have conserved and important functions in seeds. The functional conservation of homoeologous genes may result in genetic redundancy and gene dosage effects for their associated seed traits, explaining why the large deletion did not cause lethal effects or completely eliminate palmitic acid in N0304-303-3.

**Electronic supplementary material:**

The online version of this article (doi:10.1007/s00122-016-2725-z) contains supplementary material, which is available to authorized users.

## Introduction

Soybean (*Glycine max*) is a dual purpose crop. The demand for soybeans is mainly driven by the high value of oil in dietary and industrial use and the protein that makes soybean meal a valuable commodity (Clemente and Cahoon 2009). With the increasing demand for soybean oil as an industrial resource for uses such as bio-fuel and greater consumer awareness of health issues related to dietary fats (Durrett et al. 2008), development of new soybean varieties with desirable fatty acid composition becomes a critical goal for genetic improvement of soybean. Commodity soybeans accumulate about 20 % oil in their seeds, which contains on average 13 % palmitic acid (16:0), 4 % stearic acid (18:0), 20 % oleic acid (18:1), 55 % linoleic acid (18:2), and 8 % linolenic acid (18:3) (Goettel et al. 2014; Pham et al. 2010). The main enzymes and metabolic pathways responsible for producing fatty acids in oilseed species are largely described (Voelker and Kinney 2001). In soybean, great efforts have been made to identify and characterize mutant alleles and to genetically engineer genes encoding enzymes involved in fatty acid biosynthesis for the improvement of oil composition (Clemente and Cahoon 2009; Wilson 2012).

Soybean is an allotetraploid species with a complex genome. The soybean genome has undergone two whole-genome duplication events that occurred 13 and 59 million years ago (Schmutz et al. 2010). It is estimated that about 75 % of genes are retained in multiple copies. Many of the duplicated genes have conserved protein functions (Lin et al. 2010; Roulin et al. 2012). The two whole genome duplications created genetic redundancy and dosage effects for many phenotypic traits in soybean, and make genetic engineering for fatty acid composition more complicated and less predictable. Enzymatic reactions in the fatty acid metabolic pathway are often determined by a combination of more than two homoeologous genes (Pham et al. 2010). For example, the delta-twelve fatty acid desaturase 2 enzymes (FAD2) are encoded by a multi-gene family. The *FAD2*-*2* sub-family, which consists of three genes, is mainly expressed in soybean vegetative tissues while the two microsomal *FAD2*-*1* genes [*FAD2*-*1A* (Glyma10g42470) and *FAD2*-*1B* (Glyma20g24530)] are mainly expressed in developing seeds. It has been shown that soybean genotypes carrying both mutated alleles of *FAD2*-*1A* and *FAD2*-*1B* have an average of 82–86 % oleic acid content, which is significantly higher than that of soybean genotypes containing each individual mutated allele (Pham et al. 2011). Understanding the contribution of each homoeologous gene to each enzymatic activity in soybean seeds facilitates the design of effective breeding strategies towards improvement of the soybean seed fatty acid profile.

Soybean oil with lower palmitic acid content offers substantial health benefits, as consumption of palmitic acid has been shown to increase the risk of developing cardiovascular diseases. A number of soybean genotypes containing low palmitic acid levels have been identified. They provide a rich genetic resource to breed new cultivars with low palmitic acid content. Some of their underlying loci (mostly referred to as “*fap*”) have been identified. *Fap* alleles associated with reduced palmitic acid levels are *fap*_*1*_ in genotype C1726 (Cardinal et al. 2014; Erickson et al. 1988), *fap*_*3*_ in A22 (De Vries et al. 2011; Fehr et al. 1991; Schnebly et al. 1994), fap* in ELLP2 (Stijšin et al. 1998), *sop1* in genotype J3 (Rahman et al. 1996; Takagi et al. 1995) and *fap*_*nc*_ in genotype N79-2077-12 (Burton et al. 1994; Cardinal et al. 2007; Wilson et al. 2001b, c). With the exception of *fap*_*nc*_, a naturally occurring mutation, all other *fap* alleles were developed by chemical mutagenesis or by X-ray irradiation. While *fap*_*nc*_ is allelic with *fap*_*3*_, *fap1*, *fap*_*3*_, and fap* represent independent genetic loci. *sop1* is not allelic to *fap*_*1*_, and its allelism with other *fap* alleles has not been reported. The genes associated with *fap*_*1*_ and *fap*_*3*_ have been identified. *fap*_*1*_ is an allele of *KASIIIA* (Glyma09g41380) that encodes a 3-ketoacyl-ACP synthase enzyme III (Cardinal et al. 2014). A single nucleotide mutation that disrupts the exon1–intron1 splice junction of *KASIIIA* results in a truncated KASIIIA enzyme. *fap*_*3*_ is an allele of the *FATB1a* gene, which codes for a 16:0-acyl carrier protein (ACP) thioesterase enzyme (De Vries et al. 2011). *fap*_*3*_ has a non-synonymous substitution, which produces a detrimental effect on the FATB1a function. *fap*_*nc*_ is a second mutant allele of *FATB1a*. Southern, northern and cDNA analysis failed to detect *FATB1a* in genotypes containing the *fap*_*nc*_ allele, suggesting that *FATB1a* is deleted in *fap*_*nc*_ (Cardinal et al. 2007; Wilson et al. 2001a, c). However, the genome structural change underlying *fap*_*nc*_ has not been illustrated.

The ultimate goal of soybean seed fatty acid composition improvement is to develop cultivars containing a desirable fatty acid profile without negative impact on other soybean traits. For the design of an effective crossing and selection strategy, which fully utilizes the genetic resources, it is important to (1) identify and precisely define the genome structural changes underlying each mutant allele, (2) understand the functional redundancy of homoeologous genes and their contribution to fatty acid profiles, and (3) illustrate the impact of mutant alleles on other traits at molecular and systems levels. Availability of next-generation sequencing technologies enables us to sequence transcriptomes of soybean seeds, which simultaneously reveals two functional attributes of expressed genes, transcript sequence and accumulation levels (Goettel et al. 2014; Ozsolak and Milos 2011). Furthermore, comparative transcriptome analysis can effectively identify transcript sequence and expression variation of mutated genes among different germplasm. Their impact on expression of all other genes can be assessed at a systems level, which provides an insight into their potential interactions with other agronomic traits. This method is especially beneficial for the analysis of polyploid genomes since transcript sequences and accumulation levels of all homoeologous genes can be evaluated simultaneously to predict the contribution of each individual gene to their combinatorial protein activity (Goettel et al. 2014).

Recently, we applied next-generation sequencing technology to sequence seed transcriptomes from nine soybean genotypes varying in oil content and composition, and showed that genetic variation results in the expression change of thousands of genes (Goettel et al. 2014). To identify and characterize large structural genome rearrangements that affect the fatty acid composition of soybean seeds we further explored our transcriptome sequencing data. We developed a bioinformatics approach to identify several co-regulated gene clusters, and demonstrated that some of them are caused by large-scale genome segment deletions and amplifications. We identified a novel 254-kb deletion in genotype N0304-303-3 that includes the *FATB1a* gene encoding a fatty acyl-ACP thioesterase B. N0304-303-3 contains a reduced level of palmitic acid and also has altered levels of other major fatty acids (Goettel et al. 2014) (Suppl. Figure 1). We showed that the low palmitic acid content in N0304-303-3 was highly associated with the large deletion, suggesting that loss of FATB1a activity likely reduces palmitic acid in N0304-303-3. The deletion is located in a duplicated genome segment retained from the *Glycine*-specific whole genome duplication. Most genes in the deleted region shared a high similarity with their homoeologous genes in their expression patterns and protein sequences, which may have led to genetic redundancy and dosage effects of those homoeologs. We propose that loss of those genes due to the deletion event does not cause lethal and null phenotypes in N0304-303-3 because their homoeologous genes completely or partially complement their function.

## Materials and methods

### Plant genotypes and PCR analysis

Soybean [*Glycine max* (L.) Merrill] seeds for PI 90406-1, PI 92567, N69-2774, and N79-2077-12 were received from the USDA Soybean Germplasm Collection (http://www.ars-grin.gov/npgs/). Seeds for the remaining genotypes were provided by Dr. Joe Burton (USDA-ARS in Raleigh, NC). Genomic DNA was isolated from 100 mg of mature seed tissue using the DNeasy Plant Mini kit (Qiagen, Valencia, CA). PCR amplification reactions were conducted with the Expand High Fidelity Plus system from Roche (Mannheim, Germany) in a 25 µl volume containing 50 ng genomic DNA, 200 nM each primer, 2.5 mM MgCl_2_, and 200 µM deoxyribonucleotide phosphates (dNTPs). Amplification condition was 1 cycle at 94 °C for 4 min, followed by 30 cycles at 94 °C for 30 s, primer annealing temperature for 30 s, and 72 °C for 30 s. A final extension was conducted at 72 °C for 15 min. For sequencing, amplicons were cloned into vector pCR2.1 with the TOPO TA cloning kit supplied by Invitrogen (Carlsbad, CA). Cloned fragments were sequenced at the Iowa State University Biotechnology Center, Ames, IA. A primer pair was designed to amplify an 805-bp product from *G. max* N0304-303-3 in the region of chromosome 5 flanking the 254-kb deletion (forward primer GCCAGCTTAATGATCAGTTT, reverse primer TAGAACCACTCAGTGTTCTTTG, annealing temperature 57 °C). Another primer pair that amplifies a 530-bp region of *FATB1a* (Glyma05g08060) was designed to be used in conjunction with the amplification reactions above (forward primer CAAGTGGACACTTGGGTTTC, reverse primer GCCTCCGTGTTAGCTTATTC, annealing temperature 61 °C).

### Seed fatty acid composition analysis

Samples consisting of ten seeds each were ground to a powder using a small mortar and pestle under liquid nitrogen. Fatty acid methyl esters (FAMEs) of a subsample from each seed pool were prepared by acid methanolysis (Burkey et al. 2007). Frozen and ground seed tissue was heated to and held at 85 °C for 90 min in a 5 % HCl–95 % methanol solution. FAMEs were partitioned two times into hexane and transferred to 2-ml vials for analysis. The FAMEs were separated by gas chromatography using an HP 6890 GC (Agilent Technologies, Inc., Wilmington, DE) equipped with a DB-23 30 × 0.53 mm column (Agilent Technologies, Inc.). Operating conditions were 1-µl injection volume, a 20:1 split ratio, and He carrier gas flow of 6 ml min^−1^. Temperatures were 250, 200, and 275 °C for the injector, oven, and flame ionization detector, respectively. Peak areas of the chromatograms were analyzed using HP ChemStation software. Fatty acid contents, as percentages, were calculated as mg fatty acid g^−1^ oil.

### Bioinformatic analyses

Seed transcriptome sequencing data from nine genotypes previously generated in our laboratory were used in this study (Goettel et al. 2014). RNA-seq data for the nine soybean genotypes are available under NCBI-GEO series accession no. GSE56297. The transcript accumulation for each gene was normalized and indicated as Fragments Per Kilobase of transcript per Million mapped reads (FPKM) as previously described (Goettel et al. 2014). A gene with a mean FPKM value of all examined genotypes higher than 0.5 or with FPKM values higher than 0 in all examined genotypes was identified as transcribed. The normalized accumulation values of each gene were used to calculate their *Z* scores as following:

$$Z \,{\text{score}} = ({\text{x}}{-}\mu )/\sigma$$

where *x* = log2 (Sample_(FPKM + 1)_)

$$\mu = \left(\sum_{{{\text{sample 1}} \ldots {\text{sample }}n}} {\text{log2(Sample}}_{{({\text{FPKM }} + { 1})}} )\right)/n$$

where *n* is the total number of samples, *σ* is the standard deviation of *μ*.

A custom Perl script was developed to identify co-regulated genome regions within a genotype that contained four or more adjacent and transcribed genes each with a *Z* score less than or equal to −2 or more than or equal to +2. The regions identified were categorized as putative large deletions or amplifications for further validation. *Z* scores of all differentially transcribed genes were displayed as a heat map in their chromosomal gene order.

The deleted genome sequences were used as queries in BLASTN searches to identify their homoeologous regions in the soybean genome. FASTA sequence files, GFF annotation files and comparison files generated by BLASTN were used as input files for the Artemis Comparison Tool (ACT) (Carver et al. 2005) to compare the duplicated soybean sequences and analyze their syntenic relationship.

MEGA5 (Tamura et al. 2011) was used for the evolutionary comparison of all homoeologous genes in the duplicated regions. Coding sequences of homoeologous genes were first aligned by ClustalW. Numbers of synonymous and non-synonymous substitutions per site were then calculated using the Nei–Gojobori model (1986). All positions containing gaps and missing data were eliminated.

## Results

### Prediction of large segment deletions and amplifications based on transcriptome sequencing

In a recent study, we sequenced the seed transcriptomes of nine soybean genotypes that vary in oil composition and content and identified a set of transcript SNPs and small indels (Goettel et al. 2014). Here, we developed a computational method to identify co-regulated gene clusters and predict large genome segment deletions and amplifications by comparing transcriptome sequencing data from nine genotypes. In our approach, we used the *Z* score to measure expression differences among the genotypes for each gene. The genomic regions containing adjacent genes within a genotype with positive or negative *Z* score values for each transcript above or below a threshold indicates that those genes are coordinately up-regulated or down-regulated. Genomic regions containing adjacent down-regulated genes with little or no transcript accumulation are indicative of putative deletions. We applied the algorithm in the analysis of the nine genotypes and identified four genomic regions in three genotypes (M23, N0304-303-3 and R02-6268F) that each contained more than three adjacent down-regulated genes with a *Z* score value of ≤−2 (Table 1). The genomic region in M23 mapped to a previously identified 164-kb deletion on chromosome 10 (Bolon et al. 2011; Rahman et al. 1994). The two co-regulated regions in N0304-303-3 were caused by a 254-kb deletion (see below). Additionally, we identified five genomic regions in four genotypes (Jack, FAM94-41, R05-591 and R08-1450) that each contained more than three adjacent up-regulated genes with *Z* score values of more than  or equal to 2 (Table 1). The coordinately up-regulated genome region in Jack coincided with the *Rhg1* locus (for resistance to *Heteroderaglycines*), which is composed of four adjacent genes (Glyma18g02580, Glyma18g02590, Glyma18g02600 and Glyma18g02610). This finding is consistent with our previous report showing the expression of the four genes at the *Rhg1* locus was tenfold up-regulated due to a tenfold genome segment amplification in Jack (Goettel et al. 2014). Thus, this computational algorithm could be used effectively to both identify genome regions containing coordinately regulated genes and to predict large structural genome variation such as deletions and amplifications.

**Table 1:** Co-regulated gene clusters identified in nine genotypes

No. of adjacent genes	Jack	FAM94-41	M23	N0304-303-3	R02-6268F	R05-591	R08-1450	Total
Down-regulated^a^								
4	0	0	0	0	**1**	0	0	1
7	0	0	0	**2**	0	0	0	2
18	0	0	**1**	0	0	0	0	1
Total	0	0	1	2	1	0	0	4
Up-regulated^b^								
4	**1**	0	0	0	0	**1**	0	2
5	0	0	0	0	0	0	**1**	1
7	0	**1**	0	0	0	0	0	1
8	0	0	0	0	0	0	**1**	1
Total	1	1	0	0	0	1	2	5

^a^Down-regulated genes with *Z* scores ≤ −2

^b^Up-regulated genes with *Z* scores ≥2

### Discovery and characterization of a novel 254-kb genomic deletion in N0304-303-3

**Fig. 1:**
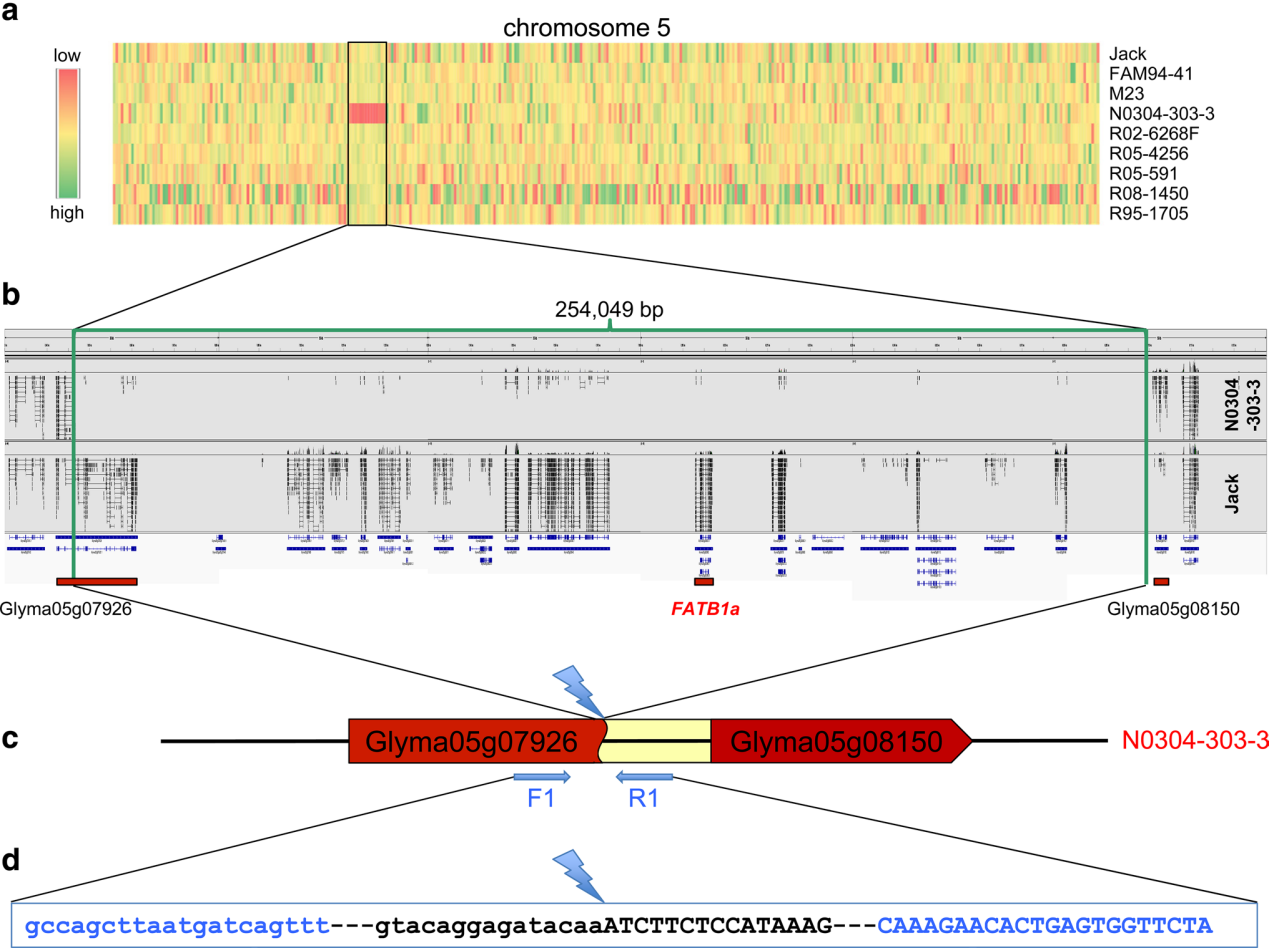
Identification of a 254-kb deletion on chromosome 5 in N0304-303-3. **a** This heat map represents expression variation among nine soybean genotypes. The *Z* score values of all genes arranged by chromosome location were displayed using a color gradient. Please note the *red cluster* of adjacent genes in N0304-303-3 that appear to be expressed at a significantly lower rate. These genes are listed in Table 2. **b** An IGV view of the region on chromosome 5, which contains the N0304-303-3 deletion, shows RNA sequence read alignments of soybean genotypes N0304-303-3 and Jack, and corresponding gene models. The deletion breakpoints, which are indicated by *green bars*, were determined by PCR analysis and sequencing of the amplification product (see below). The deletion in N0304-303-3 is 254,049 bp in size and includes 19 gene models. The *FATB1a* gene, and the genes located in or next to the breakpoints are marked by *red bars*. **c** The deletion in N0304-303-3 generated a fusion gene that consists of the 5′ end of Glyma05g07926 (in *red*), a small intergenic sequence (in *yellow*) and Glyma05g08150 (in *red*). The *blue arrows* represent both PCR primers used for the amplification across the deletion site (not drawn to scale). **d** The partial sequence of the PCR product spanning the deletion junction is presented. The primer binding sites are depicted in *blue*. The 5′ region flanking the deletion site is shown in *lower-case letters*, while the 3′ region is in *upper-case letters* (color figure online)

The two down-regulated genomic regions in N0304-303-3 were separated by one down-regulated gene with a *Z* score value of −1.7. Therefore, the two down-regulated regions likely represent two sub-regions of a single large down-regulated genome segment in N0304-303-3. This region mapped to chromosome 5 and contained 18 genes (Table 2). Fourteen of the 18 genes were expressed in soybean seeds of the other analyzed genotypes, but none of them were transcribed at a significant level in N0304-303-3, except for Glyma05g07926 located at one border of the genome region (Fig. 1a; Table 2). While a large number of RNA reads in N0304-303-3 aligned to the first eight exons of Glyma05g07926, only very few reads, which probably account for frequently seen cross-contamination during the sequencing process (Tosar et al. 2014; Zhang et al. 2012), were found in the remaining 17 exons at the 3′ end of the gene (Fig. 1b). A number of sequencing reads aligned to a 525-bp long region upstream of Glyma05g08150, which was adjacent to the other border of the genome region. In contrast to N0304-303-3, a significantly higher number of RNA reads from all other genotypes aligned to most exons in the region, but not to the intergenic sequence between Glyma05g08140 and Glyma05g08150 (Fig. 1b). Those data suggest that a large genomic segment was deleted between exon 8 of Glyma05g07926 and a short region upstream of Glyma05g08150 in N0304-303-3 (Fig. 1c).

**Table 2:** Analysis of genes in the N0304-303-3 deleted region and their homoeologs on chr17

Annotation	Homoeologous genes			Expression						Gene comparison			
	Gene pair #^a^	Genes chr5	Genes chr17	N0304-303-3 *Z* score chr5	N0304-303-3 *Z* score chr17	N0304-303-3 FPKM chr5	N0304-303-3 FPKM chr17	Mean FPKM chr5^b^	Mean FPKM chr17	Protein similarity (%)	*K*_a_^c^	*K*_s_^c^	*K*_a_/*K*_s_^c^
TBP-associated factor 2	10	Glyma05g07926	Glyma17g13080	−2.0	0.5	3.7	9.5	6.6	8.7	96.9	0.02	0.12	0.15
TCP family transcription factor	11	Glyma05g07943	Glyma17g13065	NA	NA	0.0	0.0	0.0	0.0	88.3	0.04	0.10	0.40
MUTS homolog 2	12		Glyma17g13050		1.8		7.0		4.8				
Transmembrane Fragile-X-F-associated protein	13	Glyma05g07960	Glyma17g13040	−2.6	−0.3	0.1	8.9	6.1	9.7	97.6	0.02	0.08	0.29
Unknown	14	Glyma05g07970	Glyma17g13030	−2.5	0.2	0.0	2.3	3.0	2.2	90.2	0.05	0.10	0.51
Duplicated homeodomain-like superfamily protein	15	Glyma05g07980	Glyma17g13010	−2.3	−0.5	0.2	2.3	4.7	3.8	93.5	0.02	0.06	0.29
Histone deacetylase 15	16^d^	Glyma05g07991	Glyma17g13000	−2.6	−0.6	0.2	2.5	7.4	2.7	94.9	0.04	0.11	0.33
S-adenosyl-l-methionine-dependent methyl transferases protein	18	Glyma05g08011	Glyma17g12985	−2.6	0.0	0.0	4.2	3.0	4.2	91.7	0.09	0.13	0.67
Plant protein of unknown function (DUF936)	19	Glyma05g08020	Glyma17g12970	−1.7	1.2	0.0	1.2	2.2	1.0	81.1	0.03	0.14	0.21
Unknown	20	Glyma05g08030	Glyma17g12960	−2.6	0.3	0.9	15.4	38.2	14.9	89.5	0.03	0.13	0.19
DNAJ heat shock N-terminal domain-containing protein	21	Glyma05g08040	Glyma17g12950	−2.5	1.7	0.3	20.1	12.7	11.9	95.3	0.02	0.13	0.12
Fatty acyl-ACP thioesterases B: FATB1a (chr5), FATB1b (chr17**)**	22	Glyma05g08060	Glyma17g12940	−2.6	0.3	0.1	5.5	18.3	5.2	98.3	0.02	0.09	0.18
Protein phosphatase 2A regulatory B subunit family protein	23	Glyma05g08070	Glyma17g12930	−2.6	−0.1	0.4	11.7	13.5	12.2	93.4	0.04	0.13	0.29
Unknown	24	Glyma05g08080	Glyma17g12925	NA	NA	0.0	0.0	0.0	0.0	NA	NA	NA	NA
Protein of unknown function DUF92, transmembrane	25	Glyma05g08090	Glyma17g12920	NA	−0.8	0.0	2.3	0.0	3.0	80.5	0.10	0.21	0.48
Pleiotropic drug resistance 4	26	Glyma05g08100	Glyma17g12910	NA	−0.4	0.0	0.6	0.0	0.7	97.7	0.01	0.11	0.09
LisH dimerisation motif; WD40/YVTN repeat-like-containing domain	27	Glyma05g08110	Glyma17g12900	−2.3	−1.2	0.0	0.0	1.0	0.2	84.7	0.06	0.11	0.55
Sodium bile acid symporter family	28	Glyma05g08120	Glyma17g12890	−2.4	−0.9	0.0	0.1	1.4	0.5	95.9	0.03	0.09	0.33
Leucine-rich repeat protein kinase family protein	29	Glyma05g08140	Glyma17g12880	−2.4	0.4	0.1	4.0	3.7	3.5	87.2	0.04	0.22	0.16
Average				−2.4	0.1	0.3	5.1	6.8	4.7	91.57	0.04	0.12	0.31

^a^The gene numbering corresponds to Suppl. Figure 3

^b^The mean FPKM values are based on the remaining eight soybean genotypes without N0304-303-3

^c^The substitution rates *K*_a_, *K*_s_ and their ratios for the homoeologous genes are shown

^d^Glyma05g08001 was eliminated from the table as it is likely misannotated (Glyma05g07991 and Glyma05g08001 should be merged)

To verify the putative deletion on chromosome 5, we carried out a genomic PCR analysis using primers that flank the predicted deletion site in N0304-303-3 (see Fig. 1c for approximate primer location). As expected, DNA from N0304-303-3 produced an 805-bp PCR product, while DNA from the control genotype failed to produce any PCR products because the DNA fragment between both primers was too long to be amplified. Sequencing of the PCR product revealed that a 254,049-bp long genomic region was deleted between Gm5:7,846,734 and Gm5:8,100,782. No change has been detected in the sequences flanking the deletion site (Fig. 1d).

The sequencing reads found in the intergenic region downstream of the 3′ deletion site suggest that Glyma05g07926 was transcribed across the deletion junction thereby creating a novel transcript consisting of the 5′ sequence of Glyma05g07926, the intergenic region upstream of Glyma05g08150, and Glyma05g08150 (Fig. 1c). We also identified two RNA read pairs in N0304-303-3 that spanned the large DNA deletion (data not shown), which provided additional evidence for the creation of a fusion gene. The Glyma05g07926 gene encodes a TBP-ASSOCIATED FACTOR 2, which is involved in the RNA transcription machinery. It will be interesting to determine whether the new fusion transcript has a biological function.

### The 254-kb deletion containing the *FATB1a* gene and was correlated with low palmitic acid levels in N0304-303-3 and its progenitors

**Fig. 2:**
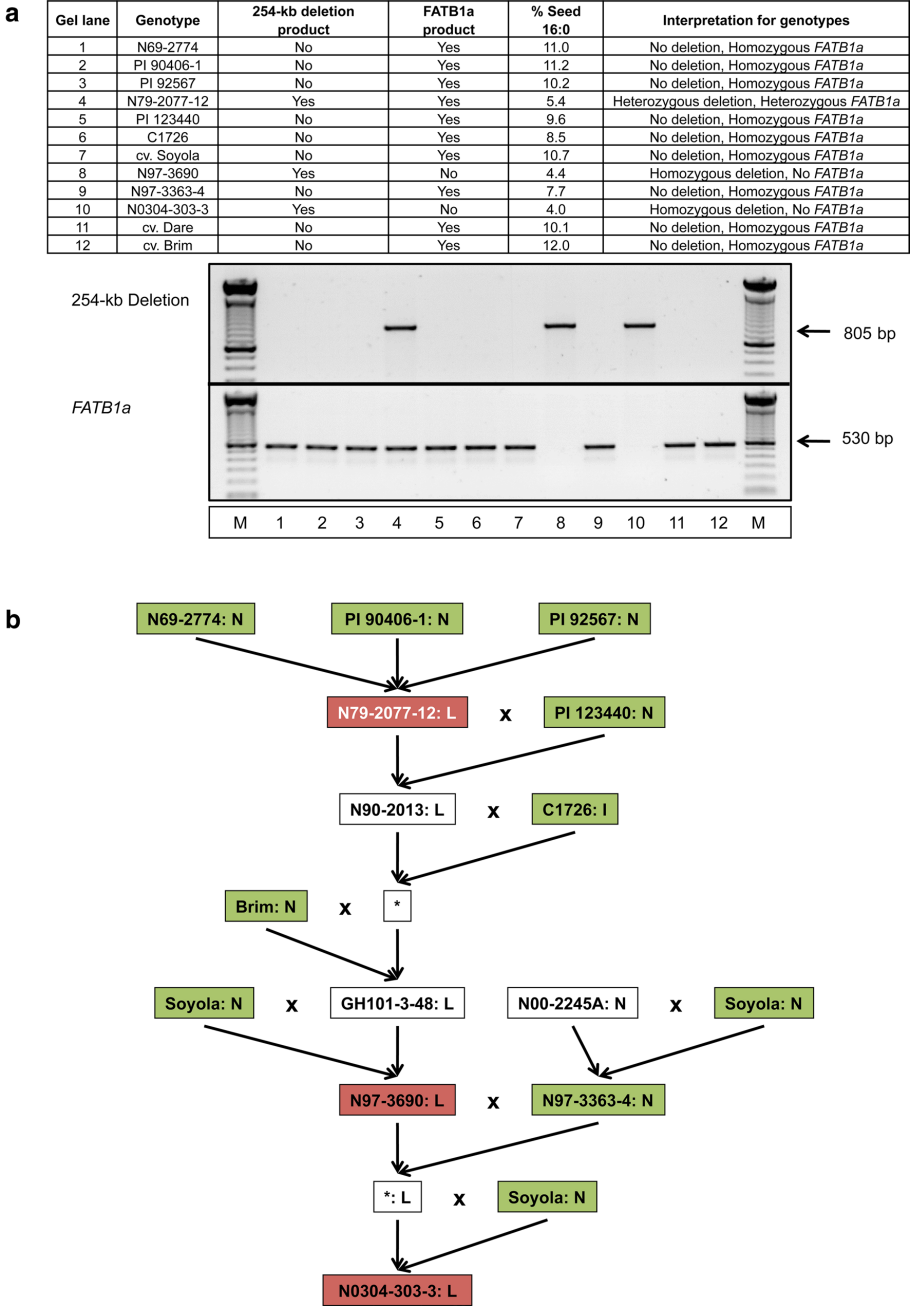
Origin of the large deletion discovered in N0304-303-3. **a** Extant soybean genotypes associated with the development of N0304-303-3 were screened by PCR for presence of the large deletion and the *FATB1a* gene. If the 254-kb deletion is present in one or both (diploid) chromosomes 5, then an 805 bp product will be amplified. If the *FATB1*a gene is present, either in haploid or diploid condition, then a 530 bp product will be amplified. The presence/absence of the deletion and *FATB1a* in homozygous or heterozygous condition was deduced from both PCR results (see summary table). **b** A pedigree of genotypes involved in the development of N0304-303-3 is presented together with their deletion genotype (see **a**) and their palmitic acid content. Genotypes in a *red box* contain the deletion, while genotypes in a *green box* lack the deletion. A *white font in a red box* indicates a genotype with a homozygous deletion, whereas a *black font in a red box* represents genotypes with a heterozygous deletion. The palmitic acid content is given after the genotype name as normal (*N*), intermediate (*I*), or a low (*L*) (color figure online)

We showed that the deleted region in N0304-303-3 contained a *FATB1a* gene (Glyma05g08060) encoding a fatty acyl-ACP thioesterase B (Fig. 2; Table 2). The enzyme hydrolyzes palmitic acid-ACP to produce palmitic acid in the de novo fatty acid biosynthetic pathway (Suppl. Figure 2) (Li-Beisson et al. 2013). Consequently, the deletion of the *FATB1a* gene might cause the reduced accumulation of palmitic acid observed in N0304-303-3. Two sets of PCR primers were designed to detect the 254-kb deletion and the *FATB1a* gene in 11 extant soybean genotypes involved in the development of N0304-303-3. PCR amplification should produce an 805-bp long DNA fragment from genotypes carrying the 254-kb deletion, and a 530-bp DNA fragment from genotypes containing the *FATB1a* gene. The PCR analysis revealed that N69-2774, PI 90406-1, PI 92567, PI 123440, C1726, cv. Soyola, N97-3363-4, and cv. Brim produced the PCR amplicon expected from the *FATB1a* gene. None of eight genotypes produced the PCR amplicon expected from the deletion (Fig. 2a), suggesting that the eight genotypes were homozygous for both presence of *FATB1a* and absence of the deletion. N79-2077-12 produced PCR products from both primer sets, indicating that the genotype contains the deletion and *FATB1a* in heterozygous condition (Fig. 2a). N0304-303-3 and N97-3690 produced the expected PCR product from the deletion-flanking region, but not for the *FATB1a* gene, indicating that they were homozygous for the 254-kb deletion. In addition, we measured the palmitic acid content in all genotypes, and showed that the 254-kb deletion in homozygous and heterozygous condition was correlated with low palmitic acid levels. Genotypes homozygous for *FATB1a* produced palmitic acid at an average level of 9.8 % (Fig. 2a), which is comparable to commodity soybean oil. The hemizygous *FATB1a* genotype (N97-2077-12) accumulated 5.4 % of palmitic acid while the homozygous deletion genotypes, N97-3690 and N0304-303-3, had an average of 4.2 %. It is possible that the zygosity status of the deletion affects the palmitic acid content. We detected the 254-kb deletion in N79-2077-12, but not in any of its three progenitors (PI 90406-1, PI 92567 and N69-2774) (Fig. 2b). This suggests that the 254-kb deletion may have occurred during the development of N79-2077-12 from those progenitor genotypes. The deletion was then most likely transmitted from genotype N79-2077-12 through N97-3690 to N0304-303-3. The PCR primers designed to amplify *FATB1a* and the sequences flanking the 254-kb deletion can be used in molecular breeding to select for a low palmitic acid phenotype in segregating populations.

**Fig. 3:**
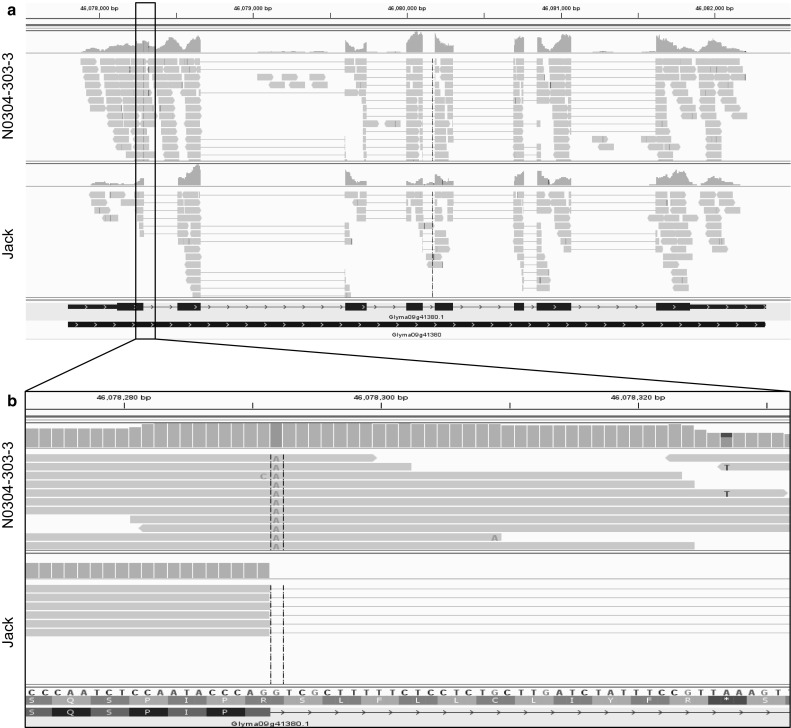
IGV view of the *KASIIIA* gene encoding a 3-ketoacyl ACP synthase. **a** RNA-seq reads for N0304-303-3 and Jack aligning to the *KASIIIA* gene model are shown. Please note that RNA-seq reads align to the first intron of N0304-303-3 while no reads can be detected in the intron for Jack. **b** A zoomed-in view of the first exon–intron junction reveals a single nucleotide change from G to A at the 5′ splice consensus sequence in N0304-303-3. The SNP may have prevented the splicing of intron 1 in N0304-303-3. Retention of the first intron results in a reading frame shift subsequently leading to a premature stop codon (shown in *red*) (color figure online)

We also observed that the first intron of the *KASIIIA* gene (Glyma09g41380) was not spliced out in N0304-303-3 (Fig. 3). This resulted in a premature stop codon that likely renders the encoded 3-ketoacyl-ACP synthase III nonfunctional. Transcript accumulation of *KASIIIA* was 1.7-fold higher in N0304-303-3 than the average of the other genotypes. The intron retained in the *KASIIIA* transcript had a nucleotide change from GT to AT in its 5′ splicing consensus sequence. The same nucleotide change was reported previously in the *fap*_*1*_ allele of C1726 (Cardinal et al. 2014). C1726 was also involved in the development of N0304-303-3. It is likely that the *KASIIIA* allele was introduced into N0304-303-3 from C1726. C1726, which does not contain the large genomic deletion containing the *FATB1a* gene, had an intermediate palmitic acid level of 8.5 % (Fig. 2a). The mutated *KASIIIA* allele potentially contributes to additional reduction of palmitic acid levels in N0304-303-3 (4.0 % of palmitic acid) compared to N79-2077-12 (5.2 % of palmitic acid). However, its effects on the accumulation of palmitic acid in the presence of the 254-kb deletion remain to be determined.

### The 254-kb deletion was located in a functionally conserved duplicated chromosome segment

The 254-kb deleted region was located on a duplicated genome segment retained from the *Glycine*-specific whole genome duplication that occurred 13 million years ago. The genes in the duplicated regions were highly syntenic (Suppl. Figure 2). Each of the 18 annotated gene models in the deleted region had a corresponding homoeologous gene in its duplicated region on chromosome 17. Seventeen of the eighteen annotated gene models in the deletion region are likely functional genes. Gene models Glyma05g08080/Glyma17g12925 shared similarities in their DNA sequence, but no protein identity. Neither of those two gene models was expressed in seeds. They were likely pseudo genes and were omitted from further analysis (Table 2).

We calculated the synonymous substitution rates *K*_s_, the non-synonymous substitution rates *K*_a_ and the *K*_a_/*K*_s_ ratio for the 17 duplicated gene pairs, which revealed high similarities in their coding sequence alignments. The *K*_s_ values of these 17 genes in the deleted regions ranged from 0.06 to 0.22 with an average of 0.13 (Table 2). These values are consistent with genes that emerged from the most recent genome duplication event 13 million years ago (Lin et al. 2010; Roulin et al. 2012; Schmutz et al. 2010). The *K*_a_/*K*_s_ ratios for the homoeologous genes ranged from 0.09 to 0.67 with an average of 0.30, which was much less than 1, suggesting that the homoeologous genes underwent a strong purifying selection. The proteins encoded by those duplicated genes had a high sequence similarity with an average of 91.6 % (Table 2), indicating that they may have identical or similar protein activities.

**Fig. 4:**
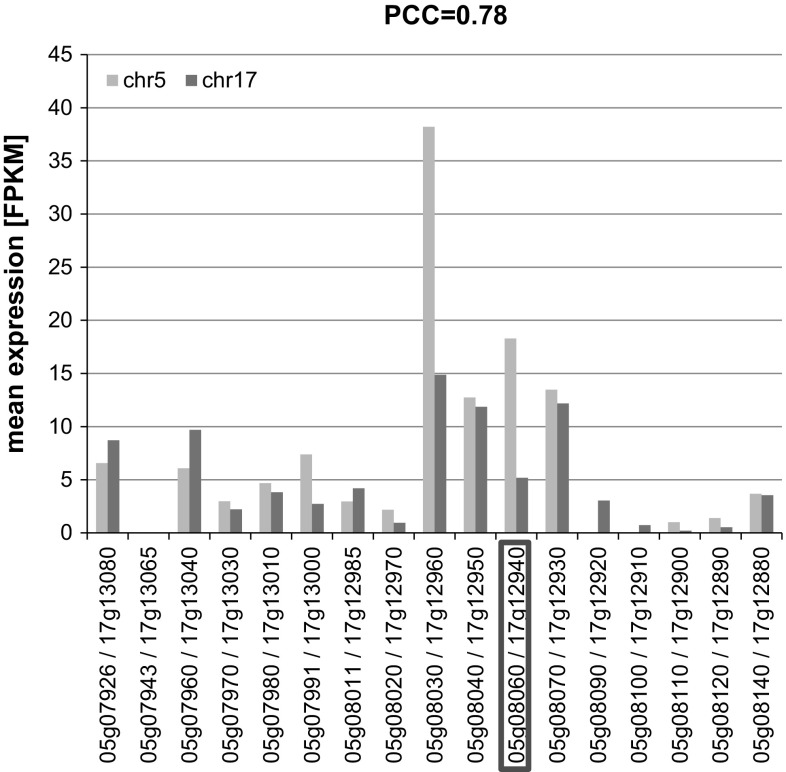
Expression of genes in the N0304-303-3 deleted region. The average expression of genes in the N0304-303-3 deleted region and their homoeologs, which are derived from eight soybean genotypes, are displayed. Gene annotations and additional information are listed in Table 2. The gene pair framed in *red* refers to *FATB1a* and *FATB1b* (color figure online)

We examined the expression of the 17 homoeologous gene pairs in soybean seeds based on our transcriptome sequencing data. Fourteen of these 17 genes in the deleted region were expressed in seeds of the other examined genotypes, which did not contain the genomic deletion (Table 2). Interestingly, all of their corresponding homoeologous genes on chromosome 17 were expressed in seeds as well. Those gene pairs were transcribed at similar levels with an overall Pearson’s correlation coefficient (PCC) value of 0.78 (Fig. 4). Three of the seventeen genes were not expressed in seeds. However, two of their homoeologous genes on chromosome 17 were expressed in seeds, suggesting that their expression patterns diverged after the whole genome duplication.

Soybean contains four genes encoding fatty acyl-ACP thioesterases B. *FATB1a* was highly expressed in soybean seeds at a level of 18.3 FPKM. Its homoeologous gene on chromosome 17, *FATB1b* (Glyma17g12940), was expressed at a lower level of 5.18 FPKM. FATB1a and FATB1b shared 98.3 % protein sequence similarity, strongly suggesting that both FATB1a and FATB1b have similar enzymatic functions. In addition, *FATB1a* and *FATB1b* had a very low *K*_a_/*K*_s_ value of 0.18, indicating that they were subject to a strong purifying selection. The other *FATB* genes, *FATB2a* (Glyma04g21910) and *FATB2b* (Glyma06g23560) were also abundantly expressed in soybean seeds at levels of 44.1 and 3.4 FPKM, respectively (Table 3). FATB1a shared 76.4 and 76.2 % protein similarity with FATB2a and FATB2b respectively, suggesting that they potentially share similar enzymatic functions with FATB1a. Thus, the deletion of *FATB1a* probably did not result in a complete loss of fatty acyl-ACP thioesterases B activities in soybean seeds. The remaining 4 % palmitic acid content observed in N0304-303-3 is likely attributed to the activities of the three functional *FATB* genes.

**Table 3:** Expression of *FATB* genes (in FPKM)

Gene	Gene ID	N0304-303-3	Mean	Stdev	(%)
*FATB1a*	Glyma05g08060	0.11	18.30	4.1	25.80
*FATB1b*	Glyma17g12940	5.52	5.14	1.3	7.24
*FATB2a*	Glyma04g21910	26.79	44.13	40.3	62.22
*FATB2b*	Glyma06g23560	2.88	3.37	2.8	4.75

### The large deletion in M23 was also located in a functionally conserved duplicated region

As predicted above, a large down-regulated gene cluster mapped to a previously reported 164-kb deletion in M23 (Bolon et al. 2011). While this deletion was previously reported, the expression of genes in the deleted region and their evolutionary relationship with their homoeologous genes has not been characterized. Like the large deletion in N0304-303-3, we observed that the deleted region in M23 was also located on a duplicated chromosome segment retained from the *Glycine*-specific whole genome duplication (Table 4; Suppl. Figure 4). Out of the 24 genes in the deleted region, 22 had homoeologs in a duplicated region on chromosome 20 (Table 4). The homoeologous gene pairs had an average *K*_a_/*K*_s_ ratio of 0.34 and their encoded proteins had an average sequence similarity of 88.95 % (Table 4), suggesting that the genes in the M23 deletion also underwent purifying selection and are most likely subject to functional constrains. Nineteen out of twenty-four genes in the M23-deleted region were expressed in seeds (Table 4). The majority of the 18 homoeologous genes were expressed in seeds at similar levels with a PCC value of 0.96 (Table 4; Suppl. Figure 5).

**Table 4:** Analysis of genes in the M23 deleted region and their homoeologs on chr20

Annotation	Homoeologous genes			Expression						Gene comparison			
	Gene pair #^a^	Genes chr10	Genes chr20	M23 *Z* score chr10	M23 *Z* score chr20	M23 FPKM chr10	M23 FPKM chr20	Mean FPKM chr10^b^	Mean FPKM chr20	Protein similarity (%)	*K*_a_^c^	*K*_s_^c^	*K*_a_/*K*_s_^c^
Inner centromere protein, ARK binding region	4	Glyma10g42420	Glyma20g24645	−0.6	−0.3	0.2	0.6	0.3	0.7	89.0	0.07	0.11	0.65
Pentatricopeptide repeat (PPR) superfamily protein	5	Glyma10g42430	Glyma20g24630	NA	−0.7	0.0	0.2	0.0	0.2	94.4	0.04	0.12	0.35
Cationic amino acid transporter 7	6	Glyma10g42440	Glyma20g24620	−1.9	1.9	0.1	5.5	5.1	2.0	86.0	0.06	0.19	0.30
Duplicated homeodomain-like	7	Glyma10g42450	Glyma20g24600	−2.1	−0.5	0.3	2.6	4.2	3.3	89.7	0.02	0.16	0.15
F1F0-ATPase inhibitor protein	8	Glyma10g42461	Glyma20g24510^d^	−2.6	0.5	7.5	200.3	145.5	174.5	70.6	0.09	0.14	0.63
Fatty acid desaturase 2: FAD2-1A and FAD2-1B	9	Glyma10g42470	Glyma20g24530^d^	−2.4	0.8	13.7	3829.9	430.7	2846.0	97.7	0.03	0.12	0.25
Vesicle-associated membrane protein 727	10	Glyma10g42480	Glyma20g24540^d^	−2.6	0.4	1.5	36.7	40.8	34.4	97.1	0.15	0.31	0.48
Clathrin adaptor complexes medium subunit	11	Glyma10g42490	Glyma20g24550^d^	−2.6	1.3	0.9	27.4	12.0	21.4	98.3	0.01	0.07	0.19
Hemerythrin HHE cation binding domain	12	Glyma10g42500	Glyma20g24560^d^	−2.5	−0.2	1.0	6.6	21.9	7.0	97.2	0.03	0.15	0.18
Polynucleotidyl transferase, RIBONUCLEASE HII-RELATED	13	Glyma10g42510	Glyma20g24570^d^	−2.5	NA	0.3	0.0	5.5	0.0	39.1	0.44	0.56	0.79
Unknown	14	Glyma10g42520	Glyma20g24580^d^	−2.3	1.2	0.1	9.4	5.1	6.7	65.1	0.08	0.12	0.70
Unknown	15	Glyma10g42525		NA		0.0		0.0					
Nucleic acid-binding, OB-fold-like protein	16	Glyma10g42530	Glyma20g24590^d^	−2.0	−0.3	0.0	2.8	2.7	3.5	97.2	0.02	0.06	0.34
Ferredoxin 3	17	Glyma10g42540	Glyma20g24500	−2.4	0.0	0.7	45.7	8.1	46.1	99.4	0.02	0.15	0.15
Phosphotyrosine protein phosphatase	18	Glyma10g42551	Glyma20g24480	−2.4	0.4	0.0	6.0	2.1	5.5	94.9	0.04	0.18	0.22
ARM repeat superfamily protein	19	Glyma10g42560	Glyma20g24470	−2.4	1.0	0.1	5.6	3.0	4.5	88.7	0.02	0.10	0.23
Unknown	20	Glyma10g42570	Glyma20g24460	NA	0.4	0.0	0.3	0.0	0.3	91.1	0.03	0.10	0.26
ACT domain-containing protein	21	Glyma10g42580	Glyma20g24450	−2.6	0.9	0.7	21.3	19.3	17.1	98.6	0.01	0.06	0.18
Unknown	22	Glyma10g42590	Glyma20g24440	−2.5	0.4	0.2	3.2	3.2	3.0	93.6	0.03	0.07	0.51
Radical SAM superfamily protein	23	Glyma10g42600	Glyma20g24430	−2.2	−0.5	0.0	3.9	1.3	4.4	85.1	0.03	0.12	0.25
Cupin	24	Glyma10g42611		NA		0.0		0.0			0.04	0.11	0.33
TRICHOME BIREFRINGENCE-LIKE 36	25	Glyma10g42621	Glyma20g24410	NA	1.2	0.0	1.3	0.0	0.7	89.5	0.06	0.13	0.44
GHMP kinase family protein	26	Glyma10g42630	Glyma20g24400	−2.6	0.6	0.3	19.3	4.9	16.6	94.5	0.02	0.12	0.17
PPR repeat	27		Glyma20g24390		NA		0.0		0.0				
20S proteasome alpha subunit E2	28	Glyma10g42650	Glyma20g24380	−2.5	1.2	6.5	18.9	20.1	16.2	100.0	0.00	0.10	0.00
Average				−2.3	0.5	1.4	184.7	30.7	139.7	88.9	0.06	0.15	0.34

^a^The gene numbering corresponds to Suppl. Figure 4

^b^The mean FPKM values are based on the remaining eight soybean genotypes without M23

^c^The substitution rates *K*_a_, *K*_s_ and their ratios for the homoeologous genes are shown

^d^A segment of eight genes is inverted on chromosome 20

The deleted region contains the *FAD2*-*1A* gene (Glyma10g42470), which encodes one of two fatty acid desaturases 2 responsible for converting oleic acid into linoleic acid in seeds. It was shown that the loss of *FAD2*-*1A* results in the mid-oleic acid phenotype of M23 (Alt et al. 2005; Sandhu et al. 2007). *FAD2*-*1A* and its homoeologous gene *FAD2*-*1B* on chromosome 20 were highly expressed in seeds. Their proteins shared 97.7 % sequence similarity (Table 4). The deletion of *FAD2*-*1A* in M23 only reduces, it does not completely abolish the ability of M23 to catalyze the desaturation of oleic to linoleic acid during fatty acid biosynthesis.

## Discussion

Genome sequencing is widely used to characterize genome structure variations. Recently, we successfully applied transcriptome sequencing technologies to identify expression variation and small-scale genome sequence changes such as SNPs and 1 or 2-bp indels in nine soybean genotypes. In this study, we identified a total of nine genomic regions containing genotype-specific co-regulated gene clusters in soybean seeds. Out of the four down-regulated genomic regions, one was mapped to a previously identified large deletion in M23. We demonstrated that two down-regulated regions in N0304-303-3 were caused by a novel 254-kb deletion. One of the up-regulated genomic regions in Jack mapped to the *Rhg1* locus, which is consistent with our previous observation that the copy number of the four genes in the *Rhg1* locus are amplified by about tenfold in Jack (Goettel et al. 2014). Thus, all of the four examined co-regulated gene clusters/genome regions were associated with large genome structural variation, demonstrating the high effectiveness of the approach to predict large-scale genome rearrangements. It will be interesting to investigate if the remaining co-regulated gene clusters also concur with large structural variation. In addition, the transcriptome sequencing approach revealed a transcribed fusion gene in N0304-303-3 that was created by the large deletion event. Collections of soybean mutants such as fast-neutron induced mutants containing large genome structural variation have been generated by the soybean community. Comparative genomic hybridization and genome sequencing have been successfully used to identify and characterize the large genome structural variation in those soybean mutants (Bolon et al. 2011). While transcriptome sequencing has been widely used to study global gene transcriptional regulation, its application for the discovery of large-scale genome structural variation in those soybean mutants could be further explored. In summary, the transcriptome sequencing approach allows us to effectively predict large-scale genome structural variation, and also provides insight into resulting transcriptional changes at the molecular and systems levels.

We showed that the 254-kb deletion was associated with a reduction of palmitic acid in N0304-303-3 and the genotypes involved in the development of N0304-303-3, suggesting that the low palmitic acid content was caused by the large deletion. The deleted region contains the *FATB1a* gene, which encodes a fatty acyl-ACP thioesterase B. This enzyme terminates the fatty acyl chain extension during de novo fatty acyl synthesis by hydrolyzing acyl groups and releasing free palmitic acid (Li-Beisson et al. 2013). Also, non-functional *FATB1a* alleles have been shown to result in lower palmitic acid levels (Cardinal et al. 2007, 2014). Therefore the loss of the FATB1a enzymatic activity is likely responsible for the decrease of palmitic acid levels in N0304-303-4. Interestingly, N79-2077-12, which carries the *fap*_*nc*_ allele, was involved in developing N0304-303-3. Our analysis showed that N79-2077-12 contained the 254-kb deletion, suggesting that the 254-kb deletion represents the molecular basis underlying the *fap*_*nc*_ allele. The large-scale deletion also explains a previous report that Southern, northern and cDNA analysis failed to detect *FATB1a* in *fap*_*nc*_ genotypes (Cardinal et al. 2007; Wilson et al. 2001a, c). The three genotypes used to develop N79-2077 lacked the 254-kb deletion and had normal palmitic acid levels. One of those genotypes, N69-2774, is an *ms1ms1* male-sterile maintainer genotype (Brim and Young 1971). Genetic crosses with it are known to induce instability during meiosis. Thus, the initial mating with N69-2774 likely caused the 254-kb deletion in developing N79-2077. The identification of the deletion junction site and the new fusion transcript created by the deletion allows us to develop molecular markers to detect the 254-kb deletion allele at both DNA and RNA levels for a variety of applications.

We also detected another low palmitic acid mutant allele, *fap*_*1*_, in N0304-303-3 in addition to the *FATB1a* deletion (Cardinal et al. 2014). We observed the missplicing of the first intron of the *KASIIIA* pre-mRNA and a single nucleotide mutation at the exon1–intron1 splice junction of  the *KASIIIA* transcript. This is consistent with the results from a previous characterization of *fap*_*1*_ in the EMS mutant genotype C1726 (Cardinal et al. 2014). C1726 was used in the development of N0304-303-3, and is probably the source of the *fap*_*1*_ allele in N0304-303-3 (Fig. 2b). We did not detect the 254-kb deletion in C1726 (Fig. 2a). C1726 contains an intermediate level of palmitic acid. Genetic analysis showed that there were significant epistatic interactions between *fap*_*1*_ and *fap*_*nc*_ (Cardinal et al. 2014). Their epistatic interaction may be caused by the fact that KASIIIA catalyzes the initial condensation reaction of acetyl-CoA and malonyl-ACP upstream of FATB1a in the fatty acid biosynthetic pathway.

Besides lower palmitic acid levels, N0304-303-3 also has a significantly reduced stearic acid and elevated oleic acid content. It may partially be caused by the *KASIIIA* mutation. However, it has previously been reported that genotypes homozygous for *fap*_*nc*_ have decreased stearic acid levels (Cardinal et al. 2007; Rebetzke et al. 1998, 2001). In *Arabidopsis*, FATB thioesterases showed significant activity with 18:1-ACP and 18:0-ACP (Salas and Ohlrogge 2002), which is consistent with the reduction of stearic acid seen in N0304-303-3. The increased oleic acid content in N0304-303-3 may be caused by the redirection from synthesizing palmitic and stearic acid into oleic acid synthesis (Upchurch and Ramirez 2010).

We showed that all four *FATB* genes encoded proteins with high sequence similarity and were expressed at significant levels in seeds, suggesting that palmitic acid production is controlled by all four *FATB* genes. As expected, the deletion of *FATB1a* did not entirely eliminate the production of palmitic acid in N0304-303-3, it did, however, reduce it by about 60 %. The remaining palmitic acid suggests that one or more of the three additional FATB thioesterases contribute to the palmitic acid production. This reduction in palmitic acid, however, was not fully correlated with the FPKM expression values from the *FATB* genes at mid-maturation stage (Table 3). Mutants for *FATB1b*, *FATB2a* and *FATB2b* are required to determine their individual contribution to the total palmitic acid content. The knowledge about the expression and structural variation of the *FATB* genes and alleles should be valuable for breeders to design crossing and selection strategies to modify palmitic acid production for new cultivar development.

The large-scale deletions in both M23 and N0304-303-3 were located in duplicated genome segments retained from the *Glycine*-specific whole genome duplication. Expression patterns and protein coding sequences of duplicated genes evolve rapidly if they are not subject to functional constraints (An et al. 1996a, b; Gu et al. 2002). All homoeologous gene pairs in both large-scale deletions had *K*_a_/*K*_s_ values smaller than 1, indicating that those genes are subject to a strong purifying selection and have conserved protein functions. Duplicated genes that acquire new functions (neo-functionalization) are the outcome of positive selection pressure, that is, their *K*_a_/*K*_s_ > 1. Interestingly, all homoeologous gene pairs in both deletions have *K*_a_/*K*_s_ values smaller than 1, indicating that these genes did not gain new functions after the *Glycine*-specific duplication event. Most of the duplicated genes in the deleted region had similar expression levels. However, since the expression of those homoeologous genes in the deleted region was only measured in seeds at the mid-maturation stage, sub-functionalization based on divergent expression patterns in other tissues and developmental stages is plausible for the homoeologous genes after the duplication event. Conservation of gene function and/or sub-functionalization in gene expression due to a strong purifying selection pressure are supported by *K*_a_/*K*_s_ ratios <1.

The functional conservation of duplicated genes could explain the lack of lethal or null phenotypes (i.e., the phenotype in the absence of redundant gene functions) in genotypes containing such large-scale deletions. Almost all genes in each large-scale deletion encoded functional proteins and were expressed at a significant level in seeds. We propose that the loss of so many functionally significant genes in such large deletions does not cause lethal or null phenotypes for M23 and N0304-303-3, because their functionally conserved homoeologous genes partially or fully complement the deleted genes. Any phenotype that is the result of gene products from two or more functionally conserved homoeologs may be subject to a gene dosage effect upon deletion of one or more individual homoeologs. For example, the deletion of the *FATB1a* gene reduces palmitic acid levels by about 60 % in N0304-303-3 but does not eliminate it. Likewise, the deletion of *FAD2*-*1A* does not completely block the conversion of oleic to linoleic acid since the linoleic acid content is only decreased by about 55 % (Suppl. Figure 1). Accordingly, both deletions described here may represent impressive examples of gene dosage effects in soybean. This hypothesis is consistent with a recent report of a 206-kb deletion in the high palmitic acid genotype J10 containing the *KASIIA* gene along with 16 other genes. That deleted region is also located on a duplicated chromosome segment. No notable growth defects were detected in J10 plants under field conditions (Anai et al. 2012).

Our observations for both deleted regions were consistent with the result from a genome-wide analysis of paralogs in soybean. A study of 8910 strictly duplicated gene pairs revealed that only a few duplicates were neo- or non-functionalized (Roulin et al. 2012). It has also been reported that the pseudogenization rate for duplicates within a 1 mb homoeologous soybean regions was less than that of single copy genes (Lin et al. 2010). This suggests that large deletions can occur and could be viable in many other duplicated genome segments. It also raises the possibility that large non-deleterious deletions derived from mutagenesis experiments may preferentially be located in duplicated genome segments and are less likely generated in single copy regions.

While in genetic theory duplicates are evolutionarily unstable, it has been shown in *Arabidopsis* and rice that higher impact non-synonymous changes occurred less frequently in duplicated genes than in single copy genes (Chapman et al. 2006). Evidentially, functional buffering between duplicates can persist for tens of millions of years (Wang et al. 2012). The retention of highly conserved homoeologous genes for 13 million years after the whole *Glycine*-genome duplication should offer an evolutionary advantage for soybean. Accordingly, the loss of a conserved homoeologous gene, as seen for example in large deletions, should come with reduced fitness. Although none of those large deletions result in detrimental phenotypic changes, they appear to have an effect on soybean fitness. In addition to the fatty acid composition changes in M23 and N0304-303-3, it has been reported that the *fap*_*nc*_ allele, which is caused by the 254-kb deletion, is associated with decreased yield and reduction in plant height (Cardinal et al. 2014). The M23 deletion appears to affect plant growth and development (unpublished). Thus, it seems that some of the deleted genes were not fully complemented by their homoeologs. Although natural selection may be of less concern for a crop species, selection by breeders will not favor the maintenance of a deletion in a soybean genotype with lower yield potential. The molecular and functional genomics characterization of the large deletions in N0304-303-3 and M23 offers an excellent example for genetic redundancy and dosage effects of homoeologous genes on agronomic traits, which are important in soybean genetics and breeding. Breeders may need to combine mutant alleles of homoeologous genes to develop cultivars with significantly improved target traits (Pham et al. 2010). Eventually, dosage effects may provide excellent opportunities for breeders to control a target trait quantitatively.

### **Author contribution statement**

WG and YQA conceived of the study, participated in the design and data analysis of the study. RGU provided seeds for various soybean genotypes. MR and RGU participated in the genomic PCR analysis and the seed fatty acid composition analysis. RGU provided important pedigree information for genotype N0304-303-3. YQA oversaw and coordinated the study. WG, YQA and RGU wrote the manuscript.

## Electronic supplementary material

Below is the link to the electronic supplementary material.

Supplementary material 1 Seed oil composition of soybean mutant genotypes. The fatty acid composition of soybean seeds is shown for soybean genotypes Jack and N0304-303-3. The major fatty acids are palmitic acid (16:0), stearic acid (18:0), oleic acid (18:1), linoleic acid (18:2), and linolenic acid (18:3). The *number before the colon* indicates the number of carbon atoms, and that after the colon, the number of double bonds in the fatty acid chain. The oil composition of Jack resembles that of commodity soybean oil [13 % palmitic acid (16:0), 4 % stearic acid (18:0), 20 % oleic acid (18:1), 55 % linoleic acid (18:2), and 8 % linolenic acid (18:3)]. The mutant genotype N0304-303-3 has a decreased palmitic and stearic acid content and increased oleic acid content. Figure adapted from Goettel et al. (2014) (PPTX 49 kb)

Supplementary material 2 Enzymatic reactions of KASIIIA, FATB and FATA. In fatty acid synthesis, the initial condensation of acetyl-CoA and malonyl-ACP to form the four-carbon product 3-ketoacyl-ACP is catalyzed by ketoacyl-ACP synthase III (KASIII). Six additional condensation reactions are required to produce palmitic acid-ACP (not shown). Palmitic acid-ACP can be hydrolyzed by the FATB thioesterase to release free fatty acids, which are exported from the plastid. Alternatively, palmitic acid-ACP can be elongated by KAS II to stearic acid-ACP. The majority of stearic acid-ACP is desaturated by the FAB2 stearoyl-ACP desaturase to form oleic acid-ACP. Both stearic acid-ACP and oleic acid-ACP can be hydrolyzed by the FATA thioesterase and to a smaller extent by the FATB thioesterase for the export from the plastid. ACP acyl carrier protein, KASIII ketoacyl-ACP synthase III, FATA fatty acyl thioesterase A, FATB fatty acyl thioesterase B, KASII ketoacyl-ACP synthase II, FAB2 stearoyl-ACP desaturase (figure adapted from Li-Beisson et al. 2013) (PPTX 66 kb)

Supplementary material 3 Synteny between chromosome 5 and 17 segments containing the *FATB1a* and *FATB1b* genes. Syntenic regions on chromosome 5 and 17 spanning more than 400 kb are shown. Genes on both chromosome segments are numbered such that syntenic genes were assigned the same number. Please see matching Table 2 for details on syntenic genes. Genes are displayed according to their transcriptional orientation on both strands. Genes that are included in the large deletion on chromosome 5 in N0304-303-3 are *colored in orange*. Their syntenic genes on chromosome 17 are *colored in light blue*. Genes highlighted in *green* represent *FATB1a* on chromosome 5 and *FATB1b* on chromosome 17. *Blue and red bands* between both chromosome segments indicate sequences of at least 80 % similarity. Sequence inversions are shown by *red bands* (PPTX 122 kb)

Supplementary material 4 Synteny between chromosome 10 and 20 segments containing the *FAD2-1A* and *FAD2-1B* genes. Syntenic regions on chromosome 10 and 20 spanning more than 230 kb are presented. Genes on both chromosome segments are numbered such that syntenic genes were assigned the *same number*. Please see matching Table 4 for details on syntenic genes. Genes are displayed according to their transcriptional orientation on both strands. Genes that are included in the large deletion on chromosome 10 in M23 are *colored in orange*. Their syntenic genes on chromosome 20 are *colored in light blue*. Genes highlighted in *green* represent *FAD2-1A* on chromosome 10 and *FAD2-1B* on chromosome 20. *Blue and red bands* between both chromosome segments indicate sequences of at least 80 % similarity. Please note the small inversion of eight genes on chromosome 20, which is shown by *red bands* (PPTX 114 kb)

Supplementary material 5 Transcriptional characterization of genes in the M23-deleted region. The average FPKM values of genes in the M23-deleted region and their homoeologs coming from eight soybean genotypes are displayed on a logarithmic scale. Please note that 1 FPKM was added to the average FPKM values since negative or zero values cannot be plotted correctly on log charts. Gene annotations and additional information can be found in Table 4. The gene pair framed in red refers to *FAD2-1A* and *FAD2-1B* (PPTX 67 kb)
