# Supplementary figures and images for: Identification and characterization of large DNA deletions affecting oil quality traits in soybean seeds through transcriptome sequencing analysis
Source: Theor Appl Genet. 2016 May 14;129:1577–93. doi: 10.1007/s00122-016-2725-z (PMC4943983; doi:10.1007/s00122-016-2725-z)

## Slide 1
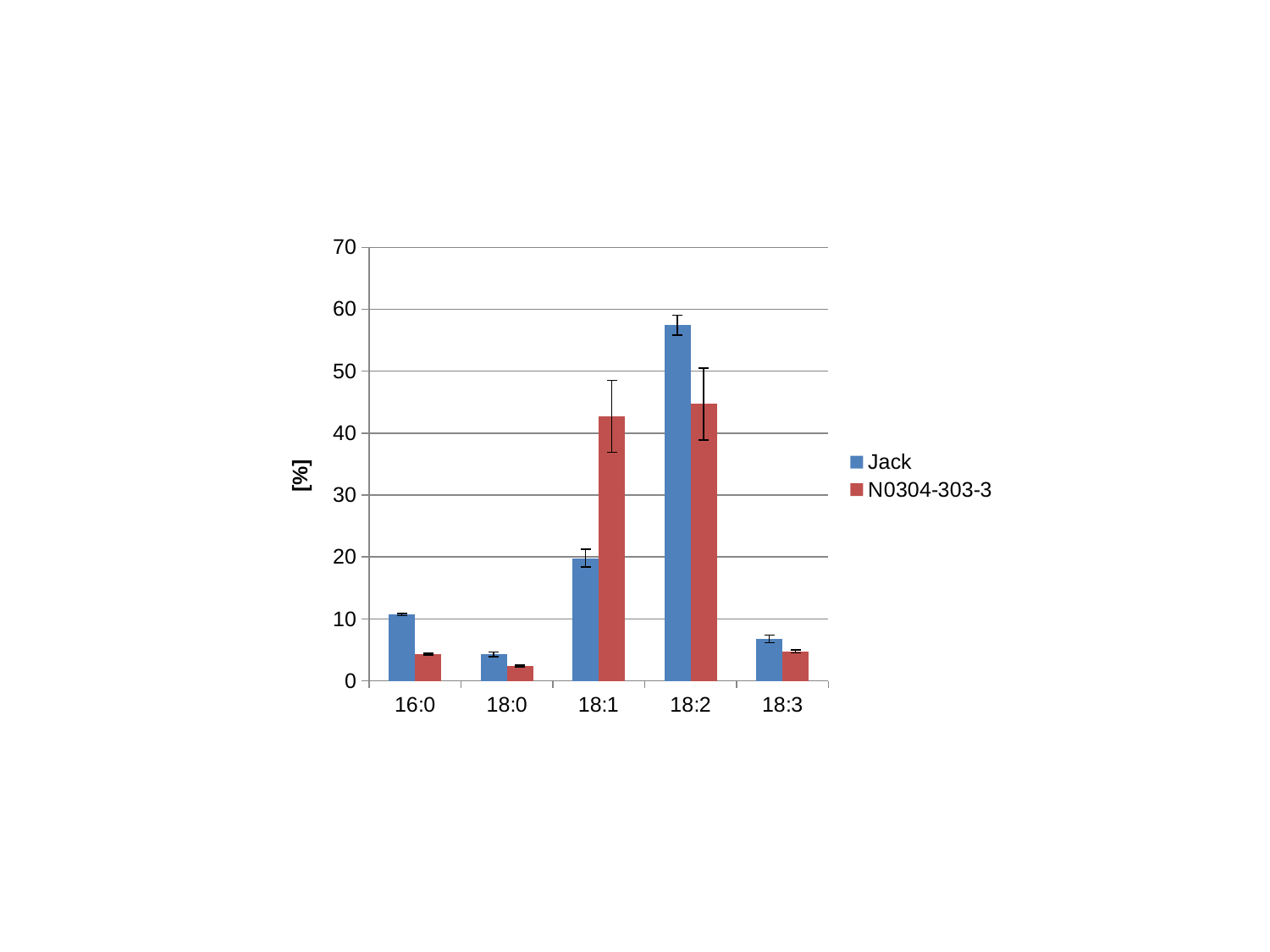

### Chart
| Category | Jack | N0304-303-3 |
|---|---|---|
| 16:0 | 10.675 | 4.306666666666667 |
| 18:0 | 4.23833333333334 | 2.398333333333333 |
| 18:1 | 19.79666666666667 | 42.72666666666656 |
| 18:2 | 57.45166666666638 | 44.71 |
| 18:3 | 6.77166666666667 | 4.748333333333332 |

Supplement: Supplementary file 1 — Supplementary material 1 Seed oil composition of soybean mutant genotypes. The fatty acid composition of soybean seeds is shown for soybean genotypes Jack and N0304-303-3. The major fatty acids are palmitic acid (16:0), stearic acid (18:0), oleic acid (18:1), linoleic acid (18:2), and linolenic acid (18:3). The number before the colon indicates the number of carbon atoms, and that after the colon, the number of double bonds in the fatty acid chain. The oil composition of Jack resembles that of commodity soybean oil [13 % palmitic acid (16:0), 4 % stearic acid (18:0), 20 % oleic acid (18:1), 55 % linoleic acid (18:2), and 8 % linolenic acid (18:3)]. The mutant genotype N0304-303-3 has a decreased palmitic and stearic acid content and increased oleic acid content. Figure adapted from Goettel et al. (2014) (PPTX 49 kb) [file 122_2016_2725_MOESM1_ESM.pptx]

## Slide 1
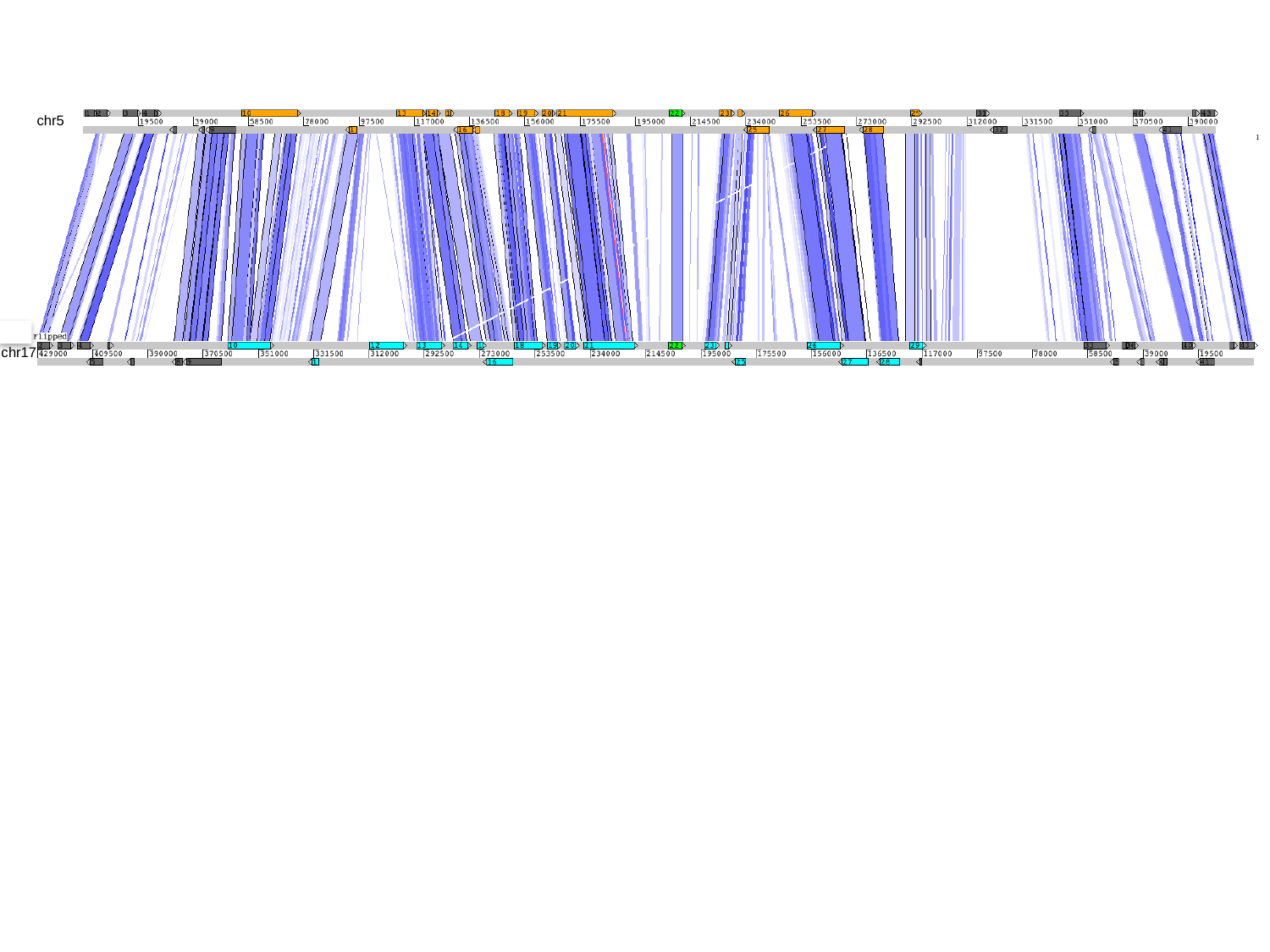

chr5
chr17

Supplement: Supplementary file 3 — Supplementary material 3 Synteny between chromosome 5 and 17 segments containing the FATB1a and FATB1b genes. Syntenic regions on chromosome 5 and 17 spanning more than 400 kb are shown. Genes on both chromosome segments are numbered such that syntenic genes were assigned the same number. Please see matching Table 2 for details on syntenic genes. Genes are displayed according to their transcriptional orientation on both strands. Genes that are included in the large deletion on chromosome 5 in N0304-303-3 are colored in orange. Their syntenic genes on chromosome 17 are colored in light blue. Genes highlighted in green represent FATB1a on chromosome 5 and FATB1b on chromosome 17. Blue and red bands between both chromosome segments indicate sequences of at least 80 % similarity. Sequence inversions are shown by red bands (PPTX 122 kb) [file 122_2016_2725_MOESM3_ESM.pptx]

## Slide 1
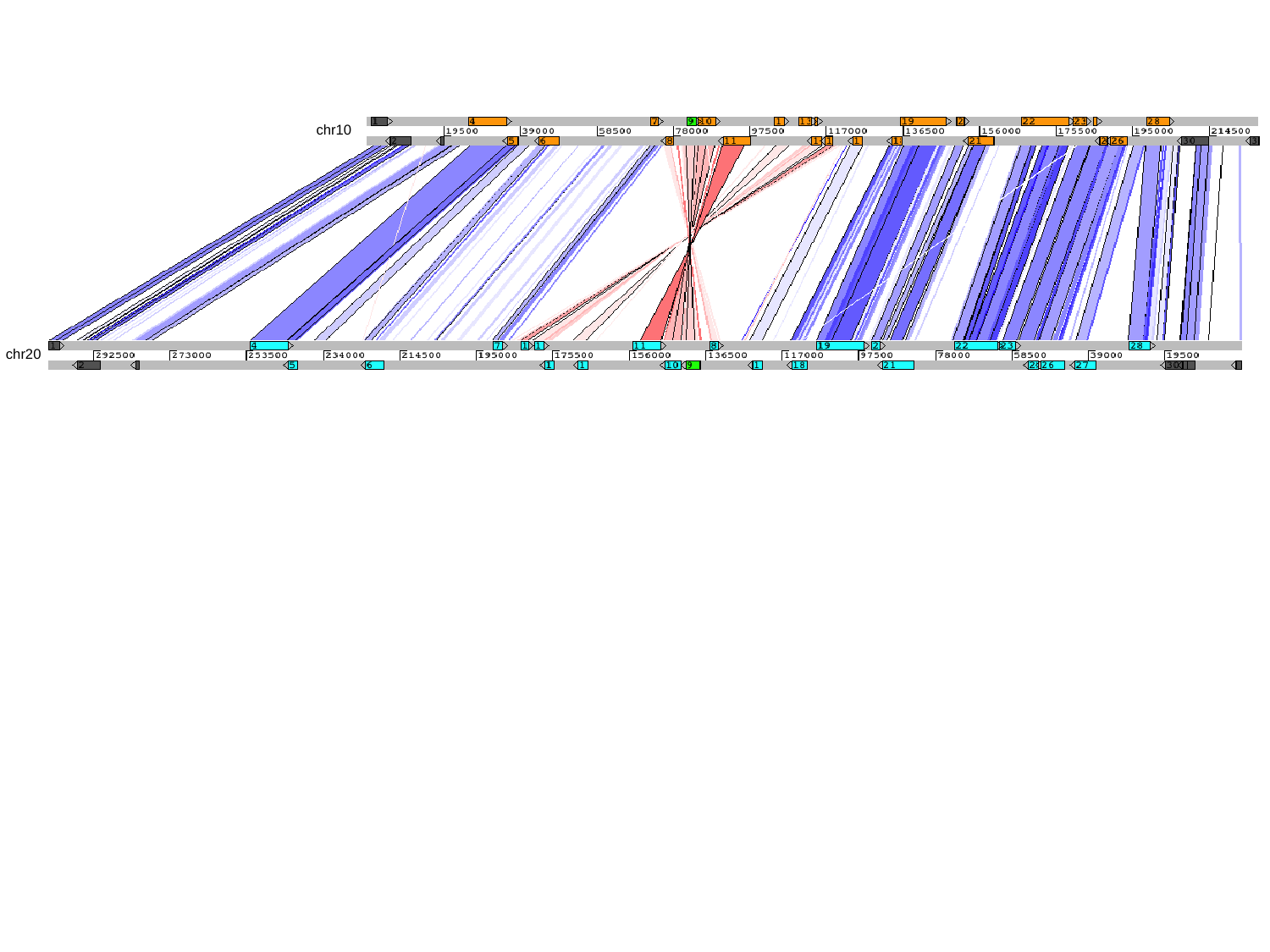

chr10
chr20

Supplement: Supplementary file 4 — Supplementary material 4 Synteny between chromosome 10 and 20 segments containing the FAD2-1A and FAD2-1B genes. Syntenic regions on chromosome 10 and 20 spanning more than 230 kb are presented. Genes on both chromosome segments are numbered such that syntenic genes were assigned the same number. Please see matching Table 4 for details on syntenic genes. Genes are displayed according to their transcriptional orientation on both strands. Genes that are included in the large deletion on chromosome 10 in M23 are colored in orange. Their syntenic genes on chromosome 20 are colored in light blue. Genes highlighted in green represent FAD2-1A on chromosome 10 and FAD2-1B on chromosome 20. Blue and red bands between both chromosome segments indicate sequences of at least 80 % similarity. Please note the small inversion of eight genes on chromosome 20, which is shown by red bands (PPTX 114 kb) [file 122_2016_2725_MOESM4_ESM.pptx]

## Slide 1
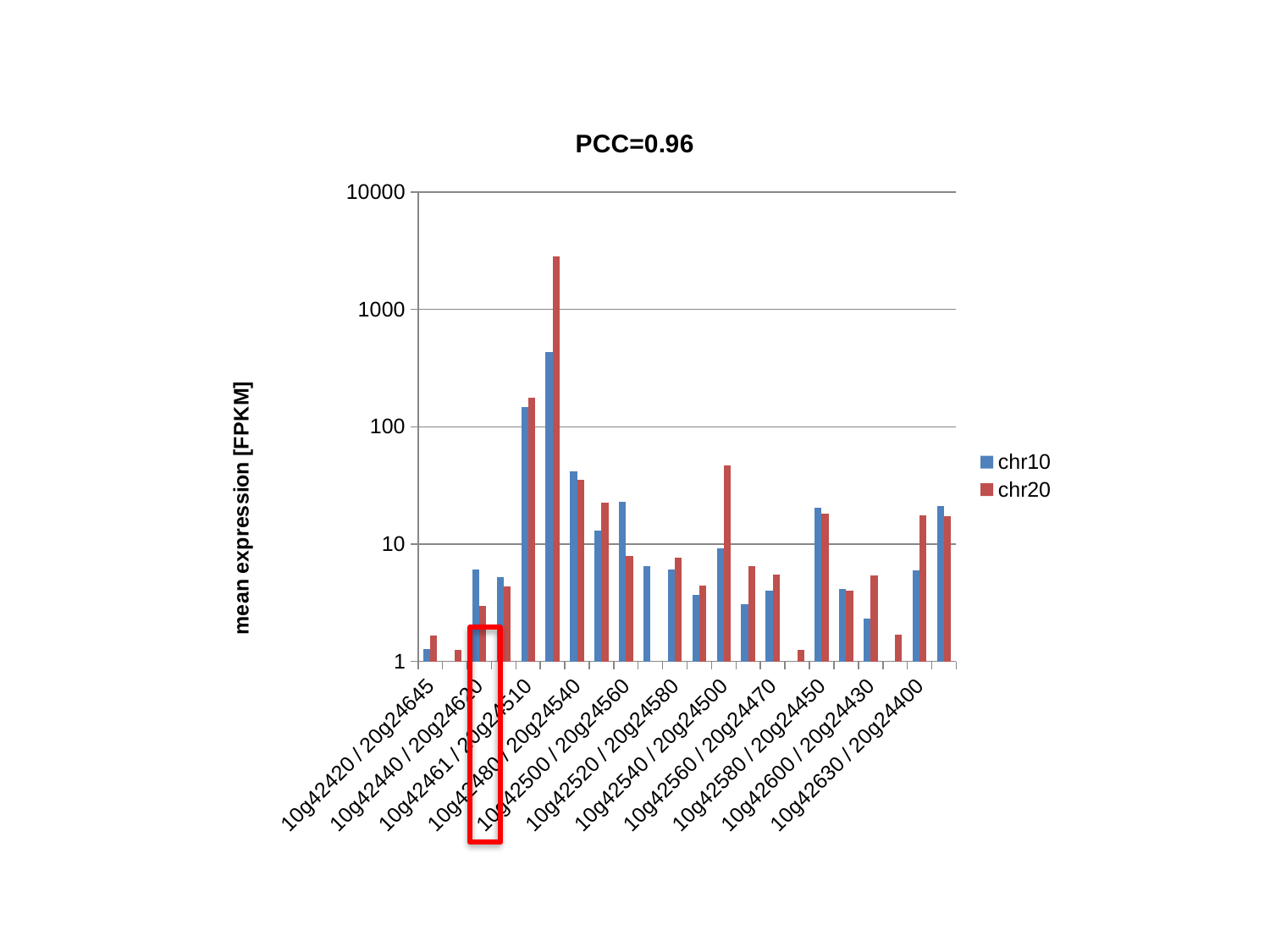

### Chart: PCC=0.96
| Category | chr10 | chr20 |
|---|---|---|
| 10g42420 / 20g24645 | 1.285307125 | 1.66446 |
| 10g42430 / 20g24630 | 1.0 | 1.24664 |
| 10g42440 / 20g24620 | 6.067576249999931 | 2.995299999999998 |
| 10g42450 / 20g24600 | 5.23039375 | 4.340929999999997 |
| 10g42461 / 20g24510 | 146.5024125 | 175.51293 |
| 10g42470 / 20g24530 | 431.7293749999992 | 2846.98889 |
| 10g42480 / 20g24540 | 41.8241125 | 35.40874 |
| 10g42490 / 20g24550 | 12.9633625 | 22.39704 |
| 10g42500 / 20g24560 | 22.92257499999998 | 7.95033 |
| 10g42510 / 20g24570 | 6.523171249999953 | 1.0 |
| 10g42520 / 20g24580 | 6.095852499999999 | 7.66246999999999 |
| 10g42530 / 20g24590 | 3.70542375 | 4.45263 |
| 10g42540 / 20g24500 | 9.1428225 | 47.14211 |
| 10g42551 / 20g24480 | 3.08977125 | 6.52325 |
| 10g42560 / 20g24470 | 4.015274999999996 | 5.528969999999997 |
| 10g42570 / 20g24460 | 1.0 | 1.25708 |
| 10g42580 / 20g24450 | 20.3293 | 18.1297 |
| 10g42590 / 20g24440 | 4.177921249999953 | 3.99779 |
| 10g42600 / 20g24430 | 2.31012275 | 5.41198 |
| 10g42621 / 20g24410 | 1.0 | 1.67682 |
| 10g42630 / 20g24400 | 5.92969875 | 17.63701 |
| 10g42650 / 20g24380 | 21.1103125 | 17.24906 |

Supplement: Supplementary file 5 — Supplementary material 5 Transcriptional characterization of genes in the M23-deleted region. The average FPKM values of genes in the M23-deleted region and their homoeologs coming from eight soybean genotypes are displayed on a logarithmic scale. Please note that 1 FPKM was added to the average FPKM values since negative or zero values cannot be plotted correctly on log charts. Gene annotations and additional information can be found in Table 4. The gene pair framed in red refers to FAD2-1A and FAD2-1B (PPTX 67 kb) [file 122_2016_2725_MOESM5_ESM.pptx]
