# Supplementary material for: Identification and characterization of large DNA deletions affecting oil quality traits in soybean seeds through transcriptome sequencing analysis
Source: Theor Appl Genet. 2016 May 14;129:1577–93. doi: 10.1007/s00122-016-2725-z (PMC4943983; doi:10.1007/s00122-016-2725-z)
Supplement: Supplementary file 2 — Supplementary material 2 Enzymatic reactions of KASIIIA, FATB and FATA. In fatty acid synthesis, the initial condensation of acetyl-CoA and malonyl-ACP to form the four-carbon product 3-ketoacyl-ACP is catalyzed by ketoacyl-ACP synthase III (KASIII). Six additional condensation reactions are required to produce palmitic acid-ACP (not shown). Palmitic acid-ACP can be hydrolyzed by the FATB thioesterase to release free fatty acids, which are exported from the plastid. Alternatively, palmitic acid-ACP can be elongated by KAS II to stearic acid-ACP. The majority of stearic acid-ACP is desaturated by the FAB2 stearoyl-ACP desaturase to form oleic acid-ACP. Both stearic acid-ACP and oleic acid-ACP can be hydrolyzed by the FATA thioesterase and to a smaller extent by the FATB thioesterase for the export from the plastid. ACP acyl carrier protein, KASIII ketoacyl-ACP synthase III, FATA fatty acyl thioesterase A, FATB fatty acyl thioesterase B, KASII ketoacyl-ACP synthase II, FAB2 stearoyl-ACP desaturase (figure adapted from Li-Beisson et al. 2013) (PPTX 66 kb) [file 122_2016_2725_MOESM2_ESM.pptx]

## Slide 1
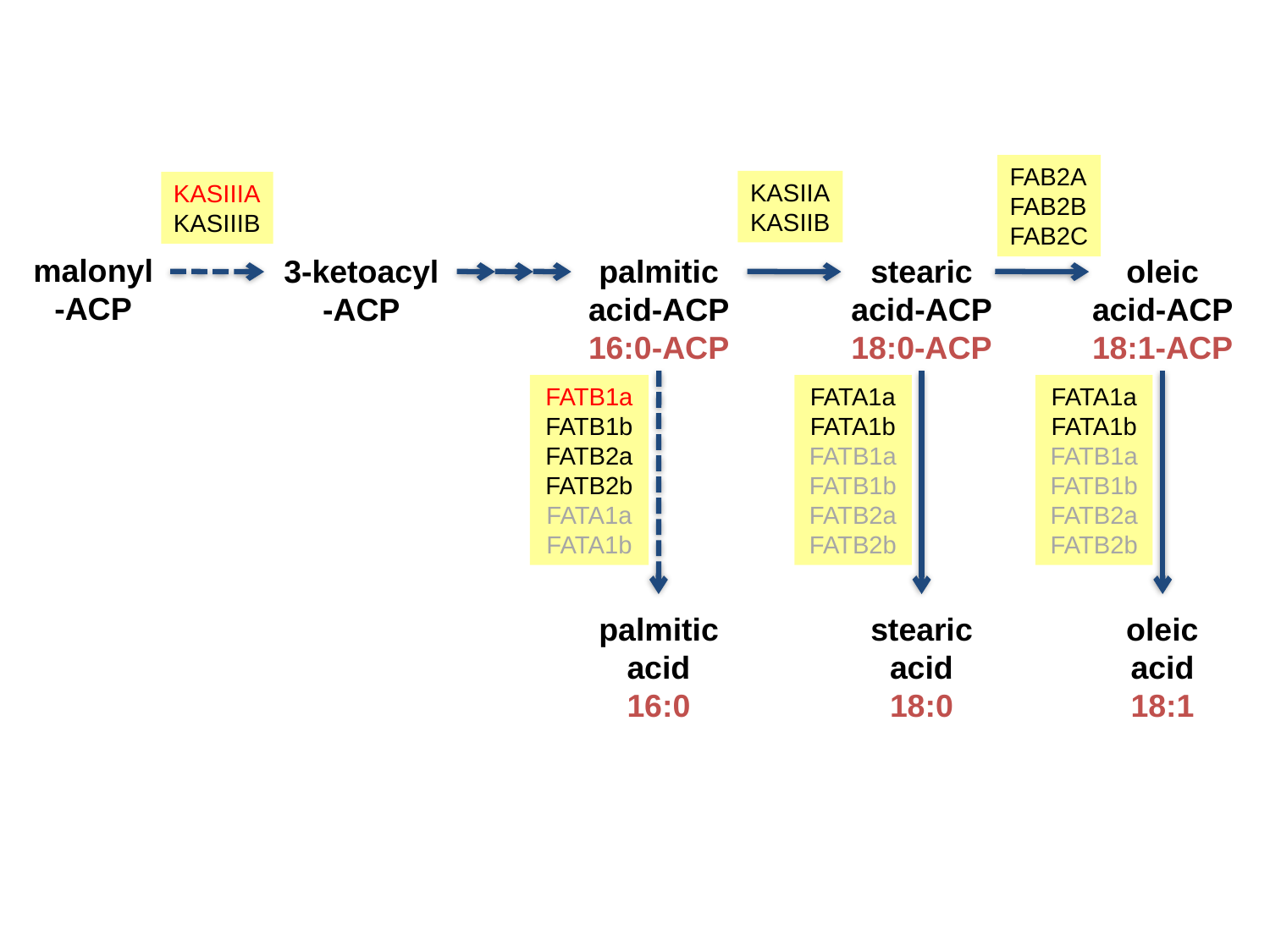

FAB2A
FAB2B
FAB2C
KASIIA
KASIIB
KASIIIA
KASIIIB
malonyl
-ACP
3-ketoacyl
-ACP
palmitic
acid-ACP
16:0-ACP
stearic
acid-ACP
18:0-ACP
oleic
acid-ACP
18:1-ACP
FATB1a
FATB1b
FATB2a
FATB2b
FATA1a
FATA1b
FATA1a
FATA1b
FATB1a
FATB1b
FATB2a
FATB2b
FATA1a
FATA1b
FATB1a
FATB1b
FATB2a
FATB2b
palmitic
acid
16:0
stearic
acid
18:0
oleic
acid
18:1
